# Characterization of Key Odor-Active Compounds in Sun-Dried Black Tea by Sensory and Instrumental-Directed Flavor Analysis

**DOI:** 10.3390/foods11121740

**Published:** 2022-06-14

**Authors:** Cong Liu, Chao Wang, Tingting Zheng, Miaomiao Zhao, Wanying Gong, Qiaomei Wang, Liang Yan, Wenjie Zhang

**Affiliations:** 1 College of Tea (Pu’ er), West Yunnan University of Applied Sciences, Pu’er 665000, China; liu_puerh@163.com (C.L.); yunnantt@126.com (T.Z.); zmm912119@163.com (M.Z.); mcfly339256486@163.com (W.G.); wqm19850127@163.com (Q.W.); yanliang879@163.com (L.Y.); 2 Pu’er Institute of Pu-erh Tea, Pu’er 665000, China; 3 Key Laboratory of Tea Science of Ministry of Education, Hunan Agricultural University, Changsha 410128, China; wangchao2809@126.com

**Keywords:** sun-dried black tea, odor-active compound, gas chromatography–olfactometry, odor activity value, aroma recombination, omission test

## Abstract

The aroma profile of sun-dried black tea (SBT) was identified by headspace solid–phase microextraction (HS–SPME) coupled with gas chromatography–mass spectrometry (GC–MS) and gas chromatography–olfactometry (GC–O). A total of 37 scents were captured by using the GC–O technique, and 35 scents with odor intensities ranging from 1.09 ± 1.93 to 9.91 ± 0.29 were identified. Twenty-one compounds were further identified as key odor-active compounds with odor activity values (OAVs) greater than or equal to one. These key odor-active compounds were restructured with their detected concentrations, and the aroma profile of the selected SBT sample was successfully imitated to a certain extent. An omission test was performed by designing 25 models and confirmed that (*E*)-*β*-damascenone, *β*-ionone, dihydro-*β*-ionone, linalool, and geraniol were the key odor-active compounds for the aroma profile of SBT. Meanwhile, phenylethyl alcohol, (*E*)-2-decenal, hexanal, and methyl salicylate were also important to the aroma profile of SBT. This study can provide theoretical support for the improvement of the aroma quality of sun-dried black tea.

## 1. Introduction

Tea has many kinds of health benefits. As a beverage, the consumption of tea ranks second only to water in the world [1]. Tea aroma is a prime criterion when evaluating tea quality, and it is also an important basis for consumers to choose. The formation of the tea aroma profile is the result of the comprehensive action of a variety of volatile compounds with different concentrations [2]. With the development and application of gas chromatography–mass spectrometry (GC–MS), more than 600 volatile compounds have been separated and identified in teas [3]. However, it is well known that not all volatile compounds have odors and contribute to the aromatic quality of finished product teas, and only a few of these compounds emit a scent and play a decisive role in the aroma profile of finished product teas, which are defined as the key odor-active compounds [4]. These odor-active compounds account for a small proportion of the complex volatile compounds, but they can dominate the overall aroma of teas. At present, how to effectively separate and identify these key odor-active compounds has become the focus of tea aroma research because the identification of key odor-active compounds in teas will have theoretical guidance for improving the aroma quality. 

Sun-dried black tea (SBT), a kind of tea that evolved from Dianhong tea, is between Dianhong and Pu-erh tea, closer to Dianhong tea [5]. SBT is made from the leaves of mature “broad leaf tea” trees (*Camellia sinensis* [L.] O. Kuntze var. *assamica* [Mast.] Kitamura) through withering, rolling, fermentation, sun-drying, and other processes [6]. The difference between the processing of SBT and Dianhong tea is that, after withering, rolling, and fermentation SBT is dried naturally by sunlight, while Dianhong tea generally takes 230–250 °C (20–30 min) to improve tea aroma, and then takes 80–100 °C to dry (50–60 min) [7]. Without the high-temperature process, the SBT retains more active substances and presents a unique sweet floral and light green aroma. Several efforts have been carried out to identify the volatile compounds in SBT in recent years [8]. However, little investigation has been carried out on the aroma characterization of SBT, and its key odor-active compounds remain unknown yet, which poses a major obstacle to the scientific elucidation of the aroma quality of the SBT.

The volatile compounds of tea are quite complicated and are found at trace levels, accounting for about 0.01% of the dry weight [9]. In particular, the results of GC–MS analysis varies greatly when different extraction methods are used, such as composition, number, relative content, and so on [10]; even with the same extraction method, the analysis results are greatly affected by the change of extraction conditions [11]. Thus, the selection of suitable extraction technique has become the decisive procedure to enhance the understanding of odor-active compounds of tea [12]. To date, the volatile extraction techniques most often used in teas include direct solvent extraction, simultaneous distillation–extraction (SDE), steam distillation-liquid/liquid extraction, Soxhlet extraction, vacuum hydrodistillation, thermal desorption, supercritical fluid extraction (SFE), solvent-assisted flavor evaporation (SAFE) and headspace solid–phase microextraction (HS–SPME) [13].

Noticeably, as one of the effective technologies to extract volatile matrices, SPME is rapidly emerging as a robust technique for the rapid, solvent-less extraction or preconcentration of volatile and semi-volatile organic compounds in a variety of scientific disciplines [14]. As an equilibrium extraction technique, the accurate quantitation of the SMPE method requires carefully controlled extraction conditions (the volume of the sample and headspace, extraction temperature, the rate of agitation, etc.). The incorporation of an internal standard into the matrix and adherence to specific sampling times will usually result in excellent quantitative correlations. The accuracy of this method has been verified in the identification of key aroma components of cranberry [15], wines [16], Kama flour [17], beers [18], Chios Mastic Gum [19], and so on. Thus, the combination of SPME and gas chromatography-olfactory (GC–O) techniques may have a great potential in the analysis of key odor-active compounds in SBT; however, no systematic research has been carried out on the odor-active compounds of SBT so far.

The main objective of this study is to explore the odor-active compounds in SBT by using instrumental (GC–O and GC–MS) analysis. Meanwhile, the odor activity value (OAVs), odor recombination and omission test were also used to confirm the key odor-active compounds of SBT. Based on these works, we can further understand the essence of the aroma profile of SBT and meanwhile can provide theoretical support for the study of the formation of key odor-active compounds of SBT.

## 2. Material and Methods

### 2.1. Materials 

In total 20 sun-dried black tea samples harvested in 2019 were collected from a tea market in Pu’er City (Pu’er, Yunnan, China) and Zhenyuan Taihe Sweet Tea Co., Ltd (Pu’er, Yunnan, China), and 11 representative samples were selected by 6 experienced assessors according to the “Methodology for Sensory Evaluation of Tea (Chinese Standards, GB/T 23776–2018)” in the sensory analysis laboratory of Yunnan Tasly Deepure Biological Tea Group Co., Ltd (Pu’er, Yunnan, China). All the representative samples were ground by a grinder with low temperature and passed through 30 mesh for future use. In order to improve the accuracy and applicability of this study, the experiment sample used in the optimization process was prepared by mixing an equal mass of 11 representative sun-dried black tea samples.

### 2.2. Reagents and Chemicals 

Hexanal (≥99%), (*E*)-2-hexenal (98.0%), benzaldehyde (>99%), 1-octen-3-ol (98%), 2-pentyl-furan (98%), *D*-limonene (≥99%), benzeneacetaldehyde (95%), linalool (98%), *L*-borneol (98%), epoxylinalol (>98%), methyl salicylate (≥99 %), *β*-cyclocitral (95%), (3*Z*)-3-hexenyl 2-methylbutanoate (98%), 1-methyl-naphthalene (98%), geranic acid (90%), n-hexyl caproate (≥98%), dihydroactinidiolide (98%), and (*E*)-nerolidol (95%) were purchased from Macklin (Shanghai, China). Linalool oxide I (99%), linalool oxide II (96%), caryophyllene (90%), geranyl acetone (99%), and n-alkanes solution (C_8_-C_32_) were purchased from J&K Chemical (Beijing, China). Hexanoic acid (≥99.5%), phenylethyl alcohol (≥99%), α-terpineol (>95%), (*Z*)-geraniol (>98%), (*E*)-geraniol (≥99%), (*E*)-2-decenal (95%), *α*-ionone (≥90%), *β*-ionone (97%) and butylated hydroxytoluene (>99.7%) were purchased from Aladdin (Shanghai, China). Safranal (90%), theaspirane (≥95%), (*E*)-*β*-damascenone (≥95%), and dihydro-*β*-ionone (≥90%) were purchased from Bidepharm (Shanghai, China). Sodium chloride (99.5%, analytical grade). Water used in this study was purified by Milli-Q purification system (Millipore, Bedford, MA, USA). 

### 2.3. HS–SPME Procedures

The 65 µm polydimethylsiloxane/divinylbenzene (PDMS/DVB) fiber (Bellefonte, PA, USA) was selected and used for HS–SPME analysis. The extraction process was established as follows: sun-dried black tea (2.0 g), sodium chloride (2.0 g), water (6.0 mL), and a microstirring bar were put into a headspace vial. The vial was sealed and placed in a 50 °C (constant temperature) water bath. After 5 min equilibrium, the fiber was inserted into the vial and kept for 60 min at 120 rpm (stirring speed). After extraction, the fiber was immediately exposed to the GC injector (250 °C, 4 min) for desorption and analysis. Before the experiment five main factors (fiber, amount of water and sodium chloride, extraction temperature, and time) were optimized, and the data of the optimization process is supplied in the Supplementary Material (Figures S1–S5).

### 2.4. GC–O and GC–MS Analysis

An Agilent 7890A GC (Agilent Technologies, Palo Alto, CA, USA) equipped with an FID and a sniffing port (Sniffer 9000, Brechbühler, Switzerland) was used for GC–O analysis. An Agilent capillary column HP-5MS (30 m × 0.25 mm i.d., 0.25 µm film thickness; Agilent Technologies, Palo Alto, CA, USA) was used for the separation of target compounds. The conditions of the GC were set as follows: initial temperature 50 °C (3 min), increased to 180 °C at a rate of 3 °C min^−1^ and maintained for 5 min, and then increased to 250 °C at a rate of 10 °C min^−1^, and maintained for 5 min; the temperature of the injection port, the injection mode and the flow velocity of carrier gas (constant-flow mode; He > 99.999%) were 250 °C, splitless, and 1.0 mL min^−1^, respectively. The conditions of olfactometry were set as follows: the flow of humidified air was 60 mL min^−1^ and the temperature of the transfer line was 250 °C. A “Y” glass was used between the FID detector and the sniff port to split the GC effluent into 1:1.

In total, 6 assessors (4 males and 2 females, average age 32, non-smoking history, odor cosmetics were not used during the experiments) were selected from Yunnan Tasly Deepure Biological Tea Group Co., Ltd (Pu’er, Yunnan, China) and Pu’er Institute of Pu-erh Tea among 17 experienced tea experts in accordance with the International Standard method [20] to perform GC–O analysis. Before the experiment, all selected assessors were trained for more than 20 h over a period of 30 d using 13 odor-active standards [*α*-terpineol, linalool, (*E*)-nerolidol, (3*Z*)-3-hexenyl 2-methylbutanoate, hexanal, 2-hexenal, methyl salicylate, phenylethyl alcohol, *α*-ionone, *β*-ionone, geraniol, (*E*)-*β*-damascenone and 1-octen-3-ol], which were detected in SBT, until all the assessors could quickly describe the aroma characteristics and aroma intensities of all selected odor compounds at their different diluted concentrations. The detection frequency method was used to analyze the aroma characteristics and aroma intensities of each effluent in GC–O analysis. A 9-point scale from 1 to 9, wherein “1”, “5”, and “9” indicated the weak odor, moderate odor, and extreme odor intensity, respectively, was used to quantify odor intensities of the effluents. During the experiments, the odor characteristics and odor intensities of the effluents were recorded and scored, respectively. Finally, the odor intensity value of each detected odor-active compound was averaged for all the scores that were detected by six assessors (three times by each assessor). After GC–O analysis, all the samples were analyzed by using GC–MS under the same capillary column, and the perceived scents were identified by the retention indices (calculated by using n-alkanes from C_8_ to C_32_) between the GC–O and the GC–MS, the mass spectra database (NIST11.L) of GC–MS, the odor characteristic of each odor-active compound, and corresponding standard of each odor-active compound. 

The conditions of GC–MS were listed as follows: an Agilent 7890A GC equipped with 5975C mass selective detector quadrupole MS instrument (Agilent Technologies, Palo Alto, CA, USA) was used to identify the perceived odor-active compounds. The conditions of GC (injector temperature, oven temperature program, the kind of carrier gas, and the flow rate of carrier gas) and the model capillary column were the same as described under GC–O analysis. The electron-impact (EI), interface temperature, ion source temperature, quadrupole temperature, mass scan range, and solvent delay time were 70 eV, 280 °C, 230 °C, 150 °C, 35–450 aum, and 3.0 min, respectively.

### 2.5. Quantitation of Odor-Active Compounds and OVA Calculation

Considering that non-volatile components may affect the aroma emission, a deodorized tea (odor-blank matrix) was prepared according to the method of the previous report [21] with minor modification during the quantitative process of odor-active compounds. The preparation process was as follows: 100 g SBT and 800 mL ultrapure water were weighed into a 2000 mL rotary evaporation bottle, following which the tea odor-active compounds were rotary evaporated in a 60 °C water bath. The process was repeated many times (greater than or equal to 5 times) until the sensory flavor of the tea was very light. Thereafter, the SBT was dried using an oven at 60 °C. The deodorized SBT, internal standard (n-decanol), and corresponding standards of odor-active compounds were used to obtain the quantitative result of each target compound. The process was listed as follows: initially, the authentic standards were dissolved using anhydrous alcohol and then diluted with ultrapure water to different concentrations. The dissolved standards dilution, internal standard (0.1 mL n-decanol, 4.15 µg/mL), and deodorized tea (2.0 g) were placed into a 20 mL headspace vial (Agilent Technologies, Palo Alto, CA, USA) and sealed. The vial was kept for 24 h at 4 °C and prepared with the HS–SPME method, as mentioned above (Section 2.3.). The quantitative standard curves of target compounds were established by plotting the peak area ratio against the corresponding concentration ratio. 

Odor activity value (OAV) is often used to evaluate the contributions of odor-active compounds and further screen the odor-active compounds that play a decisive role in the aroma profile of the analyzed samples [22]. The OAVs of compounds are equal to the ratio of corresponding concentrations to their respective odor thresholds in water, and compounds with an OAV greater than or equal to one are generally considered to contribute significantly to the aroma profile of the analyzed samples [23]. In this study, the odor thresholds of detected odor-active compounds were obtained from literature or books (References [1–17], supplied in Supplementary Material).

### 2.6. Aroma Recombination and Omission Test

For the aroma recombination experiment, the deodorized tea (treatment process is consistent with 2.5) was used as a blank matrix. The odor-active compounds with OAVs of greater than or equal to one that detected in a randomly selected SBT were added into the deodorized tea according to their detected concentrations and mixed well. The recombination sample was sealed and kept for 24 h at 4 °C before sensory analysis.

The contributions of a single odor-active compound and a group of odor-active compounds were evaluated using an omission test. Twenty-five aroma models were prepared, and the aroma characteristic and aroma intensities of the reduced models were evaluated against two complete recombination samples with a triangle test according to the method of a reported study [24]. All the recombination samples were coded with random three-digit numbers, and all the assessors were asked to select different ones through sensory analysis.

### 2.7. Descriptive Sensory Analysis

A total of 10 experienced assessors (6 males and 4 females, average age 30, with non-smoking history, odor cosmetics were not used during the experiments) were selected from Yunnan Tasly Deepure Biological Tea Group Co., Ltd (Pu’er, Yunnan, China) and Pu’er Institute of Pu-erh Tea based on the International Standard method [20] to perform the descriptive sensory analysis of SBT and recombination samples. The procedure of descriptive sensory analysis referenced the method described by the previous study with some modifications [7]. In total, 6 odor terms (floral, fruity, green/grass, sweet, woody, and unpleasant flavor) of SBT and corresponding authentic reference standard (detected in SBT; linalool for a floral note, (*Z*)-3-hexenyl isovalerate for a fruity note, hexanal for green/grass odors, n-hexyl caproate for a sweet note, *β*-ionone for a woody note) were determined by 20 assessors through 3 preliminary sessions before the sensory analysis. A 9-point scale from 1 to 9 was used, wherein “1”, “5”, and “9” indicated the weak odor, moderate odor, and extreme odor intensity, respectively. 

The descriptive sensory analysis was performed according to the “Methodology for Sensory Evaluation of Tea (Chinese Standards, GB/T 23776–2018)”. SBT (3.0 g) was placed in a special teapot and infused with 150 mL of freshly boiled water (kept for 5 min before sensory analysis). All the samples were coded with three-digit numbers and randomly provided to the assessors after brewing. The intensity value of each odor term was the average scores from 10 assessors (3 times by each assessor).

### 2.8. Statistical Analysis

Data analysis and figure drawing were performed by using MS Excel 2010 and OriginPro software (version 9.5.1, OriginLab Inc., Northampton, MA, USA), respectively.

## 3. Results and Discussion

### 3.1. GC–MS Results for Sun-Dried Black Tea

**Figure 1:**
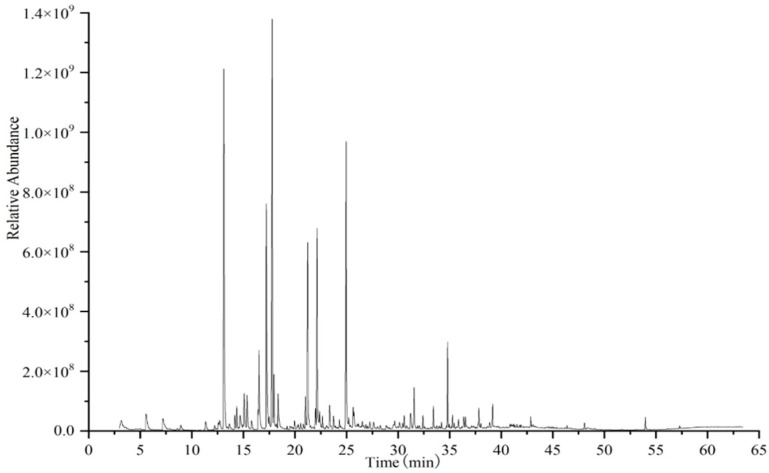
The representative total ion chromatography of sun-dried black tea.

Prior to GC–O analysis, the volatile compounds in 11 representative SBT samples were analyzed by HS–SPME/GC–MS, and the representative total ion chromatography of SBT was presented in Figure 1. A total of 86 volatile compounds were tentatively identified according to their mass spectrum and RI, including 21 hydrocarbons, 17 alcohols, 14 aldehydes, 13 ketones, 10 esters, 3 organic acids, 2 phenols, 2 ethers, 1 lactone, 1 furan, 1 nitrogen compound, and 1 oxygen heterocyclic compound, which were listed in Table S1. It was reported that 16 hydrocarbons, 17 alcohols, 11 aldehydes, 13 ketones, 10 esters, 3 organic acids, 1 ether, 1 lactone, 2 furans, and 2 nitrogen compounds were detected in a previous study [8]. The main volatile compounds in our study were consistent with those reported by Lv et al. [8].

### 3.2. Odor-Active Compounds Determination Using GC–O

In order to gain deep insights into the odor characteristics of the volatile compounds and further select the key odor-active compounds from the volatile compounds, GC–O analysis was performed. A total of 37 scents were perceived by GC–O in 11 SBT samples, and 35 compounds were identified using GC–MS based on their mass spectrum, *RIs*, odor characteristics, and authentic standards, and 2 compounds with moderate odor were not identified (Table 1). Among these detected compounds, *β*-ionone (AI: 9.91 ± 0.29), (*E*)-*β*-damascenone (AI: 9.64 ± 0.48), linalool (AI: 8.64 ± 0.98), linalool oxide II (AI: 8.45 ± 0.78), epoxylinalol (AI: 7.91 ± 0.79), linalool oxide I (AI: 7.82 ± 0.57), dihydro-*β*-ionone (AI: 7.55 ± 0.99), geraniol (AI: 7.36 ± 1.55), methyl salicylate (AI: 6.82 ± 1.11), *α*-ionone (AI: 6.36 ± 0.98), phenylethyl alcohol (AI: 5.64 ± 0.77), geranyl acetone (AI: 5.64 ± 0.97), and (*E*)-nerolidol (AI: 5.45 ± 1.87) had higher aroma intensities, which indicated that these compounds played an important role in the formation of SBT aroma. α-Ionone, *β*-ionone and dihydro-*β*-ionone, a group of carotenoid-derived aroma compounds, all presented “woody” and “violet-like” scents, which were detected in sun-dried Pu-erh tea of ancient tea plants from Bulang Mountain [25]. (*E*)-*β*-Damascenone presented “rose-like” and “sweet” scents, with low human odor perception threshold, it was reported that (*E*)-*β*-damascenone had a great contribution to the aroma profile of black teas [26]. Linalool presented pleasant “floral” and “citrus-like” scents, which were not only detected as key odor-active compounds in green tea, white tea, yellow tea, oolong tea, black tea, and dark tea samples [27] but also detected in flowers and spice plants [28,29]. Linalool oxides (linalool oxide I, linalool oxide II and epoxylinalol) presented “floral” and “woody” scents, which were detected with a high concentration in semi-fermented and fermented teas [27]. Geraniol, phenylethyl alcohol, and (*E*)-nerolidol all presented a “rose-like” scent, which was detected in many kinds of tea samples. Geraniol and (*E*)-nerolidol were reported as the key odor-active compounds for the quality of black teas [30], and (*E*)-nerolidol was also considered to be one of the key odor-active compounds for the high-quality of oolong tea [31]. Geranyl acetone presented “rose-like” and “green” scents, which were detected in black and ripened Pu-erh teas, and were considered to play an important role in the aroma profile of black and ripened Pu-erh teas [32,33]. Methyl salicylate presented “mint” and “wintergreen-like” scents, which were detected only in the teas that had a least a medium-degree fermentation but could not be detected in the green and lightly fermented teas, and it was reported that methyl salicylate and (*E*)-2-hexenal together could be used as an index to differentiate semi- and fully-fermented teas [34].

### 3.3. Quantitation of Odor-Active Compounds and OVA Calculation

The odor-active compounds from the bulk of volatile compounds of SBT were identified by using a unique technique of odor-active compounds screening. To deeper evaluate the aroma contributions of these perceived odor-active compounds, their contents were quantitated in 11 SBT samples based on internal standard and their corresponding standards, and the quantitative results of these odor-active compounds supplied in Table 2 and Table S2. The major odor-active compounds of SBT were linalool (2124.78 ± 696.93 µg/kg), linalool oxide II (1113.75 ± 302.78 µg/kg), epoxylinalol (973.71 ± 371.22 µg/kg), methyl salicylate (786.55 ± 409.55 µg/kg), geraniol (523.28 ± 459.73 µg/kg), linalool oxide I (509.49 ± 170.87 µg/kg), phenylethyl alcohol (362.70 ± 167.55 µg/kg), *β*-ionone (292.96 ± 43.00 µg/kg), benzeneacetaldehyde (259.48 ± 204.12 µg/kg), (*E*)-nerolidol (207.37 ± 79.16 µg/kg), dihydroactinidiolide (199.13 ± 93.87 µg/kg), (*E*)-*β*-damascenone (123.08 ± 46.69 µg/kg), geranyl acetone (107.28 ± 20.18 µg/kg), and theaspirane (102.94 ± 35.94 µg/kg). In contrast, *D*-limonene (34.64 ± 22.01 µg/kg), (3*Z*)-3-hexenyl 2-methylbutanoate (32.96 ± 22.46 µg/kg), (*Z*)-geraniol (31.80 ± 13.19 µg/kg), *L*-borneol (27.69 ± 26.93 µg/kg), (*E*)-2-decenal (25.56 ± 52.14 µg/kg), geranic acid (25.13 ± 38.39 µg/kg), dihydro-*β*-ionone (24.86 ± 7.32 µg/kg), and 1-octen-3-ol (23.52 ± 49.27 µg/kg) in SBT samples were relatively low. These odor-active compounds were the first quantified in SBT.

The contributions of odor-active compounds to the aroma profile of SBT not only depend on its content but also depends on its odor threshold value. Odor-active compounds with OAVs greater than or equal to one were usually considered to play a major role in the aroma profile of the samples [35]. To further deeply elucidate the contribution of the odor-active compounds in SBT, their OAVs were calculated using the quantitative results and reported thresholds, and the calculation results are shown in Table 2 and Table S3. 

The OAVs of (*E*)-*β*-damascenone (OAV: 61538.79), *β*-ionone (OAV: 41851.78), dihydro-*β*-ionone (OAV: 24856.37), linalool (OAV: 354.13), *α*-ionone (OAV: 168.68), geraniol (OAV: 163.53), phenylethyl alcohol (OAV: 74.02), (*E*)-2-decenal (OAV: 63.89), methyl salicylate (OAV: 49.16), benzeneacetaldehyde (OAV: 41.19), (*E*)-nerolidol (OAV: 20.74), linalool oxide II (OAV: 18.56), 1-octen-3-ol (OAV: 15.68), 1-methyl-naphthalene (OAV: 11.60), *β*-cyclocitral (OAV: 11.58), hexanal (OAV: 7.71), 2-pentyl-furan (OAV: 7.07), linalool oxide I (OAV: 5.09), *D*-limonene (OAV: 3.46), *α*-terpineol (OAV: 2.52), and geranyl acetone (OAV: 1.79) were greater than 1, and indicated that these compounds played an important role in the aroma profile of SBT. Among them, alcohols, ketones, hydrocarbons, and aldehydes occupied most of the components, and the results of this study were consistent with those previously reported [8]. However, the OAVs calculation showed that an odor-active compound with higher content did not mean a larger OAV or vice versa. For example, dihydro-*β*-ionone (24.86 ± 7.32 µg/kg) was present at low concentrations in SBT samples, but its OAV was 24856.37 (its threshold was 0.001 µg/kg in water). By contrast, epoxylinalol (973.71 ± 371.22 µg/kg) was present at a relatively high concentration in SBT samples, but its OAV was only 0.32. In general, the results of OAVs and GC–O were basically consistent with each other.

### 3.4. Aroma Recombination and Omission Test

**Figure 2:**
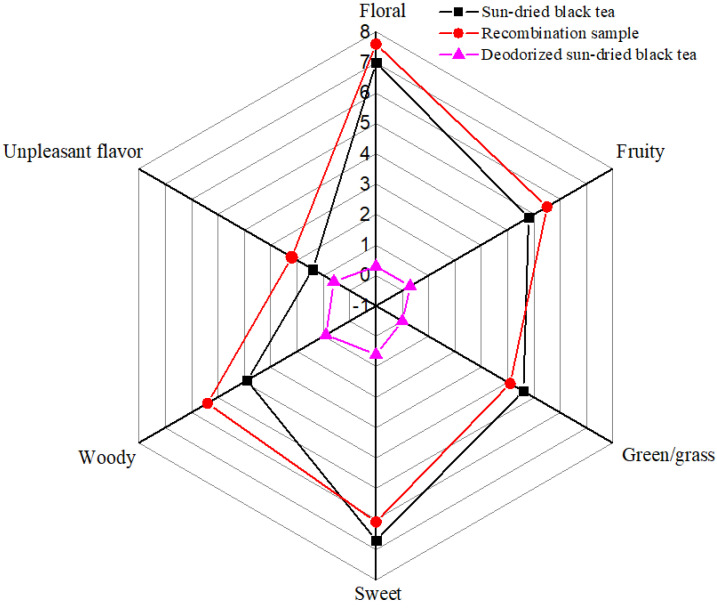
Aroma profile of the recombination sample and the sun-dried black tea.

To confirm the contributions of these compounds with OAVs greater than or equal to one, the aroma recombination test was designed and performed. The deodorized SBT was used as a blank matrix. An SBT sample was randomly selected as a reference for aroma recombination. Then, 21 compounds with OAVs greater than 1 were added to the deodorized tea based on the quantitative results of the reference sun-dried black tea and mixed well. The recombination sample was sealed and kept for 24 h at 4 °C before sensory analysis. The recombination sample and selected reference sun-dried black tea were evaluated by 10 experienced assessors according to the “Methodology for Sensory Evaluation of Tea (Chinese Standards, GB/T 23776–2018)” described in Section 2.7. Six odor terms, including floral, fruity, green/grass, sweet, woody, and unpleasant flavor were identified as the main aroma characteristics based on the sensory results of SBT samples. The sensory results of the recombination sample and selected reference sample are shown in Figure 2. The results showed that the odor profile of the SBT could be successfully imitated to a certain extent by combining these 21 odor-active compounds at their detected concentrations in the selected reference sample, specifically in terms of its floral, fruity, green/grass, and sweet scents. The recombination sample was more inclined to a woody scent. This phenomenon may be due to the conversion of the non-volatile components while preparing the deodorized matrix or the difference in the extraction amounts of odor-active compounds while brewing (the odor-active compounds of the recombination sample were easier to give off). Overall, the results of odor recombination further verified the accuracy of the identified key odor-active compounds in SBT.

In order to deeply verify the contributions of a single odor-active compound with OAVs greater than or equal to 1 to the aroma profile of SBT, the omission test was performed by designing 25 models, in which a single odor-active compound or a group of odor-active compounds was omitted. Taking the recombination sample as a reference, 25 omission models were evaluated by triangle test, and the results are supplied in Table 3. 

Model 1 showed that 10 assessors were able to perceive the entire omission of all alcohols (including linalool, geraniol, phenylethyl alcohol, (*E*)-nerolidol, linalool oxide II, 1-octen-3-ol, linalool oxide I, and *α*-terpineol) with a very high significance (*p* ≤ 0.001), indicating that these alcohols played a major role in the aroma formation of SBT. Compared with the recombination sample, the single omission of linalool (model 1-1), geraniol (model 1-2), or phenylethyl alcohol (model 1-3) showed a very high significant difference (*p* ≤ 0.001), a high significant difference (*p* ≤ 0.01), and significant difference (*p* ≤ 0.05), respectively. While the single omission of (*E*)-nerolidol (model 1-4), linalool oxide II (model 1-5), 1-octen-3-ol (model 1-6), linalool oxide I (model 1-7), or *α*-terpineol (model 1-8) showed no significant difference (*p* > 0.05), indicating that the effect of these compounds on the aroma profile of SBT is relatively small.

Model 2 showed that 10 assessors were able to perceive the entire omission of all ketones (including (*E*)-*β*-damascenone, β-ionone, dihydro-β-ionone, α-ionone, and geranyl acetone) also with a very high significance (*p* ≤ 0.001). The single omission of (*E*)-*β*-damascenone (model 2-1), β-ionone (model 2-2), or dihydro-*β*-ionone (model 2-3) also showed a very high significant difference (*p* ≤ 0.001). The single omission of *α*-ionone (model 2-4) was resulted in a significant difference (*p* ≤ 0.05). While the single omission of geranyl acetone (model 2-5) showed no significant difference (*p* > 0.05). 

Model 3 showed that 8 assessors were able to detect the entire omission of all aldehydes ((*E*)-2-decenal, benzeneacetaldehyde, *β*-cyclocitral, hexanal) with a high significance (*p* ≤ 0.01). The single omission of (*E*)-2-decenal (model 3-1) or hexanal (model 3-4) also showed a significant difference (*p* ≤ 0.05) compared with the recombination sample. While the single omission of benzeneacetaldehyde (model 3-2) or *β*-cyclocitral (model 3-3) showed no significant difference (*p* > 0.05).

Model 4 showed that only 5 assessors were able to detect the entire omission of all hydrocarbons (1-methyl-naphthalene and D-limonene) with no significant difference compared with the recombination sample. Model 5 showed that 7 assessors were able to detect the single omission of methyl salicylate with a significant difference (*p* ≤ 0.05). Model 6 showed that only 3 assessors were able to detect the single omission of 2-pentyl-furan with no significant difference (*p* > 0.05).

In summary, (*E*)-*β*-damascenone, *β*-ionone, linalool, dihydro-*β*-ionone, gerniol, phenylethyl alcohol, *α*-ionone, (*E*)-2-decenal, hexanal, and methyl salicylate were recognized as the most important key odor-active compounds in this study that are essential for the aroma quality of SBT.

## 4. Conclusions

The odor profile of sun-dried black tea was successfully identified by sensory and instrumental-directed flavor analysis. A total of 37 scents were perceived under GC–O analysis, and 35 compounds with odor intensities ranging from 1.09 ± 1.93 to 9.91 ± 0.29 were identified. Twenty-one of them were further identified as key odor-active compounds with OAVs greater than or equal to one based on their quantitative results and thresholds. These key odor-active compounds were restructured with their detected concentrations in a reference sample, and the aroma profile of the selected sun-dried black tea sample was successfully imitated to a certain extent. An omission test was performed by designing 25 models and confirmed that linalool, dihydro-*β*-ionone, *β*-ionone, and geraniol were the key odor-active compounds for the aroma profile of sun-dried black tea. Meanwhile, phenylethyl alcohol, (*E*)-*β*-damascenone, (*E*)-2-decenal, hexanal, and methyl salicylate were also important to the aroma profile of sun-dried black tea.

## Figures and Tables

**Table 1:** Odor-active compounds detected by GC-O and GC-MS in sun-dried black tea samples (11 samples).

No.	Time (min)	RI	Odor-Active Compounds	Odor Descriptors	Aroma Intensity	Frequency	Identification
1	3.84	800	Hexanal	Green, grass	3.55 ± 1.23	14	MS/RI/Odor/STD
2	5.325	851	2-Hexenal	Green, fruity	3.00 ± 1.54	13	MS/RI/Odor/STD
3	8.92	960	Benzaldehyde	Almond-like	3.00 ± 1.71	11	MS/RI/Odor/STD
4	9.69	982	1-Octen-3-ol	Mushroom-like	2.73 ± 2.00	9	MS/RI/Odor/STD
5	10.05	992	2-Pentyl-furan	Fruity	3.55 ± 0.089	12	MS/RI/Odor/STD
6	11.02	1015	Hexanoic acid	Unpleasant odor	2.64 ± 1.72	8	MS/RI/Odor/STD
7	11.56	1027	D-Limonene	Lemon-like	3.82 ± 1.58	11	MS/RI/Odor/STD
8	12.43	1046	Benzeneacetaldehyde	Sweet, rose-like	4.00 ± 1.76	12	MS/RI/Odor/STD
9	13.64	1072	Linalool oxide I	Floral, woody	7.82 ± 0.057	18	MS/RI/Odor/STD
10	14.39	1089	Linalool oxide II	Floral, woody	8.45 ± 0.78	18	MS/RI/Odor/STD
11	15.13	1105	Linalool	Floral, citrus-like	8.64 ± 0.98	18	MS/RI/Odor/STD
12	15.75	1119	Phenylethyl alcohol	Sweet, rose-like	5.64 ± 0.77	16	MS/RI/Odor/STD
13	17.90	1168	L-Borneol	Woody	2.09 ± 1.83	7	MS/RI/Odor/STD
14	18.47	1181	Epoxylinalol	Floral, woody	7.91 ± 0.79	18	MS/RI/Odor/STD
15	19.13	1196	α-Terpineol	Floral, fruity	4.27 ± 2.34	10	MS/RI/Odor/STD
16	19.29	1199	Methyl salicylate	Mint, wintergreen-like	6.82 ± 1.11	18	MS/RI/Odor/STD
17	19.43	1202	Safranal	Herbaceous, sweet	2.36 ± 2.53	6	MS/RI/Odor/STD
18	20.38	1222	β-Cyclocitral	Mint, fruity	3.09 ± 1.56	10	MS/RI/Odor/STD
19	20.92	1234	(*Z*)-Geraniol	Rose-like	3.64 ± 1.49	12	MS/RI/Odor/STD
20	21.05	1237	(3*Z*)-3-Hexenyl 2-methylbutanoate	Fruity	2.27 ± 1.48	8	MS/RI/Odor/STD
21	22.19	1261	Geraniol	Rose-like	7.36 ± 1.55	18	MS/RI/Odor/STD
22	22.35	1264	(*E*)-2-Decenal	Orange-like	1.09 ± 1.93	4	MS/RI/Odor/STD
23	23.62	1291	1-Methyl-naphthalene	Earthy-like	3.36 ± 1.92	10	MS/RI/Odor/STD
24	23.82	1295	Theaspirane	Woody, Fruity	4.91 ± 0.51	13	MS/RI/Odor/STD
25	27.51	1377	Geranic acid	Sweet, woody	1.18 ± 1.69	4	MS/RI/Odor/STD
26	27.67	1381	(*E*)-β-Damascenone	Rose-like, sweet	9.64 ± 0.48	17	MS/RI/Odor/STD
27	27.87	1385	n-Hexyl caproate	Sweet, fruity	3.36 ± 2.01	10	MS/RI/Odor/STD
28	29.02	1412	Caryophyllene	Floral, woody	3.09 ± 1.97	10	MS/RI/Odor/STD
29	29.51	1425	α-Ionone	Woody, violet-like	6.36 ± 0.98	15	MS/RI/Odor/STD
30	29.94	1435	Dihydro-β-ionone	Woody, violet-like	7.55 ± 0.99	16	MS/RI/Odor/STD
31	30.63	1453	Geranyl acetone	Rose-like, green	5.64 ± 0.97	15	MS/RI/Odor/STD
32	31.93	1485	β-Ionone	Woody, violet-like	9.91 ± 0.29	18	MS/RI/Odor/STD
33	33.00	1513	Butylated hydroxytoluene	Unpleasant odor	3.27 ± 0.75	13	MS/RI/Odor/STD
34	33.60	1528	Dihydroactinidiolide	Unpleasant odor	4.36 ± 1.49	16	MS/RI/Odor/STD
35	35.08	1566	(*E*)-Nerolidol	Rose-like	5.45 ± 1.87	16	MS/RI/Odor/STD
36	27.22	1348	Unknown1	Floral, sweet	4.03 ± 0.92	13	----
37	29.25	1417	Unknown2	Fruity, sweet	3.29 ± 0.44	12	----

**Table 2:** The results of quantitative and OAVs calculation of odor-active in sun-dried black tea samples (11 samples).

No.	Time (min)	RI	Odor-Active Compounds	The Range of Concentration (µg/kg)	Average Concentration (µg/kg)	OTs (µg/kg)	Literature ^a^	The Range of OAV	Average OAV	Identification
1	27.67	1381	(*E*)-*β*-Damascenone	58.86–238.97	123.08 ± 46.69	0.002	[1,2]	29427.88–119483.38	61538.79 ± 23342.77	MS/RI/Odor/STD
2	31.93	1485	*β*-Ionone	244.72–409.08	292.96 ± 43.00	0.007	[3]	34960.64–58440.00	41851.78 ± 6143.54	MS/RI/Odor/STD
3	29.94	1435	Dihydro-*β*-ionone	12.39–42.08	24.86 ± 7.32	0.001	[4]	12385.95–42081.44	24856.37 ± 7322.65	MS/RI/Odor/STD
4	15.13	1105	Linalool	900.49–3196.25	2124.78 ± 696.93	6	[5]	150.08–532.71	354.13 ± 116.16	MS/RI/Odor/STD
5	29.51	1425	*α*-Ionone	38.70–113.52	67.47 ± 20.43	0.4	[6]	96.75–283.81	168.68 ± 51.07	MS/RI/Odor/STD
6	22.19	1261	Geraniol	79.47–1324.51	523.28 ± 459.73	3.2	[3]	24.83–413.91	163.53 ± 143.67	MS/RI/Odor/STD
7	15.75	1119	Phenylethyl alcohol	205.78–773.33	362.70 ± 167.55	4.9	[7]	42.00–157.82	74.02 ± 34.19	MS/RI/Odor/STD
8	22.35	1264	(*E*)-2-Decenal	0.00–147.92	25.56 ± 52.14	0.4	[8]	0.00–369.80	63.89 ± 130.35	MS/RI/Odor/STD
9	19.29	1199	Methyl salicylate	212.96–1418.74	786.55 ± 409.55	16	[9]	13.31–88.67	49.16 ± 25.60	MS/RI/Odor/STD
10	12.43	1046	Benzeneacetaldehyde	0.00–680.02	259.48 ± 204.12	6.3	[10]	0.00–107.94	41.19 ± 32.40	MS/RI/Odor/STD
11	35.08	1566	(*E*)-Nerolidol	0.00–284.47	207.37 ± 79.16	10	[11]	0.00–28.45	20.74 ± 7.92	MS/RI/Odor/STD
12	14.39	1089	Linalool oxide II	853.47–1853.65	1113.75 ± 302.78	60	[10]	11.22–30.89	18.56 ± 5.05	MS/RI/Odor/STD
13	9.69	982	1-Octen-3-ol	0.00–178.23	23.52 ± 49.27	1.5	[10]	0.00–118.82	15.68 ± 32.84	MS/RI/Odor/STD
14	23.62	1291	1-Methyl-naphthalene	0.00–298.42	86.97 ± 94.37	7.5	[11]	0.00–39.79	11.60 ± 12.58	MS/RI/Odor/STD
15	20.38	1222	*β*-Cyclocitral	0.00–123.52	57.92 ± 36.36	5	[12]	0.00–24.70	11.58 ± 7.27	MS/RI/Odor/STD
16	3.845	800	Hexanal	23.65–164.09	77.13 ± 41.29	10	[10]	2.36–16.41	7.71 ± 4.13	MS/RI/Odor/STD
17	10.05	992	2-Pentyl-furan	17.29–69.98	42.43 ± 15.31	6	[13]	2.88–11.66	7.07 ± 2.55	MS/RI/Odor/STD
18	13.64	1072	Linalool oxide I	352.21–1000.30	509.49 ± 170.87	100	[10]	3.52–10.00	5.09 ± 1.71	MS/RI/Odor/STD
19	11.56	1027	*D*-Limonene	0.00–82.23	34.64 ± 22.01	10	[6]	0.00–8.22	3.46 ± 2.20	MS/RI/Odor/STD
20	19.13	1196	*α*-Terpineol	0.00–113.30	52.36 ± 35.11	20.8	[14]	0.00–5.45	2.52 ± 1.69	MS/RI/Odor/STD
21	30.63	1453	Geranyl acetone	59.66–131.61	107.28 ± 20.18	60	[6]	0.99–2.19	1.79 ± 0.34	MS/RI/Odor/STD
22	5.32	851	2-Hexenal	0.00–140.54	65.95 ± 45.45	90	[10]	0.00–1.56	0.73 ± 0.51	MS/RI/Odor/STD
23	33.61	1528	Dihydroactinidiolide	0.00–356.32	199.13 ± 93.87	500	[10]	0.00–0.71	0.40 ± 0.19	MS/RI/Odor/STD
24	17.9	1168	*L*-Borneol	0.00–82.63	27.69 ± 26.93	80	[15]	0.00–1.032	0.35 ± 0.34	MS/RI/Odor/STD
25	18.47	1181	Epoxylinalol	0.00–1626.95	973.71 ± 371.22	3000	[10]	0.17–0.54	0.32 ± 0.12	MS/RI/Odor/STD
26	23.82	1295	Theaspirane	41.06–184.22	102.94 ± 35.94	1000	[11]	0.04–0.18	0.10 ± 0.04	MS/RI/Odor/STD
27	19.43	1202	Safranal	0.00–256.32	79.54 ± 103.00	1000	[11]	0.00–0.26	0.08 ± 0.10	MS/RI/Odor/STD
28	8.92	960	Benzaldehyde	0.00–156.01	47.00 ± 45.35	750.89	[10]	0.00–0.21	0.06 ± 0.06	MS/RI/Odor/STD
29	11.02	1015	Hexanoic acid	0.00–148.17	50.05 ± 41.92	890	[10]	0.00–0.17	0.06 ± 0.05	MS/RI/Odor/STD
30	20.92	1234	(*Z*)-Geraniol	8.79–50.56	31.80 ± 13.19	680	[16]	0.01–0.05	0.05 ± 0.02	MS/RI/Odor/STD
31	29.02	1412	Caryophyllene	0.00–229.91	74.78 ± 74.52	1500	[17]	0.00–0.15	0.05 ± 0.05	MS/RI/Odor/STD
32	33.01	1513	Butylated hydroxytoluene	22.96–66.99	43.58 ± 10.80	1000	[9]	0.02–0.07	0.04 ± 0.01	MS/RI/Odor/STD
33	27.87	1385	n-Hexyl caproate	0.00–126.64	75.51 ± 45.88	6400	[11]	0.00–0.02	0.01 ± 0.01	MS/RI/Odor/STD
34	21.05	1237	(3*Z*)-3-Hexenyl 2-methylbutanoate	0.00–60.83	32.96 ± 22.46	10,000	[11]	0.00–0.01	<0.01	MS/RI/Odor/STD
35	27.51	1377	Geranic acid	0.00–117.92	25.13 ± 38.39	10,000	[11]	0.00–0.01	<0.01	MS/RI/Odor/STD

a: Odor thresholds in water, which were found in the literatures (References [1–17] are supplied in Supplementary Material).

**Table 3:** The results of the omission test.

Model	Odor-Active Compounds Omitted from the Recombination Sample	No. ^a^	Significance ^b^
1	All alcohols	10	***
1-1	Linalool	10	***
1-2	Geraniol	8	**
1-3	Phenylethyl alcohol	7	*
1-4	(*E*)-Nerolidol	5	
1-5	Linalool oxide II	5	
1-6	1-Octen-3-ol	5	
1-7	Linalool oxide I	4	
1-8	*α*-Terpineol	3	
2	All ketones	10	***
2-1	(*E*)-*β*-Damascenone	10	***
2-2	*β*-Ionone	10	***
2-3	Dihydro-*β*-ionone	10	***
2-4	*α*-Ionone	7	*
2-5	Geranyl acetone	2	
3	All aldehydes	8	**
3-1	(*E*)-2-Decenal	7	*
3-2	Benzeneacetaldehyde	5	
3-3	*β*-Cyclocitral	3	
3-4	Hexanal	7	*
4	All hydrocarbons	5	
4-1	1-Methyl-naphthalene	4	
4-2	*D*-Limonene	3	
5	Methyl salicylate	7	*
6	2-Pentyl-furan	3	

a: Number of correct judgments from 10 assessors; b: * significant (*p* ≤ 0.05); ** highly significant (*α* ≤ 0.01); *** very highly significant (*p* ≤ 0.001).

## Data Availability

Data is contained within the article.

## Supplementary Materials

The following supporting information can be downloaded at: https://www.mdpi.com/article/10.3390/foods11121740/s1, Figure S1: The extraction efficiency of four fibers; Figure S2: Influence of water amount on the number and sum of intensity value of aroma-active compounds; Figure S3: Influence of NaCl amount on the number and sum of intensity value of aroma-active compounds; Figure S4: Influence of extraction temperature on the number and sum of intensity value of aroma-active compounds; Figure S5: Influence of extraction time on the number and sum of intensity value of aroma-active compounds; Table S1: Volatile compounds detected by GC-MS in sun-dried black tea; Table S2: The quantitative results of odor-active compounds; Table S3: The OAVs of odor-active compounds.

## Abbreviations

SBT	Sun-dried black tea
HS–SPME	Headspace solid–phase microextraction
GC–O	Gas chromatography–olfactometry
GC–MS	Gas chromatography–mass spectrometry
OAV	Odor activity value
