# Supplementary material for: Characterization of Key Odor-Active Compounds in Sun-Dried Black Tea by Sensory and Instrumental-Directed Flavor Analysis
Source: Foods. 2022 Jun 14;11(12):1740. doi: 10.3390/foods11121740 (PMC9222254; doi:10.3390/foods11121740)
Supplement: Supplementary file 1 [file foods-11-01740-s001.zip › foods-1721177-Supplementary.pdf]

Table S1 Volatile compounds detected by GC-MS in sun-dried black tea

No	Time (min)	Volatile compounds	RI	Identification
1	3.845	Hexanal	800	MS/RI
2	5.325	2-Hexenal	851	MS/RI
3	5.62	4-Methyl-1-pentanol	861	MS/RI
4	6.655	5-Methyl-2-hexanol	897	MS/RI
5	8.92	Benzaldehyde	960	MS/RI
6	9.695	1-Octen-3-ol	982	MS/RI
7	9.95	6-Methyl-5-hepten-2-one	989	MS/RI
8	10.05	2-Pentyl-furan	992	MS/RI
9	11.025	Hexanoic acid	1015	MS/RI
10	11.42	o-Cymene	1024	MS/RI
11	11.565	D-Limonene	1027	MS/RI
12	11.84	2,2,6-Trimethyl-cyclohexanone	1033	MS/RI
13	12.06	(E)-β-Ocimene	1038	MS/RI
14	12.43	Benzeneacetaldehyde	1046	MS/RI
15	12.55	1-Ethyl-1H-pyrrole-2-carbaldehyde	1048	MS/RI
16	13.32	(E)-2-Hexenoic acid	1065	MS/RI
17	13.64	Linalool oxide I	1072	MS/RI
18	14.39	Linalool oxide II	1089	MS/RI
19	15.13	Linalool	1105	MS/RI
20	15.245	Hotrienol	1108	MS/RI
21	15.755	Phenylethyl alcohol	1119	MS/RI
22	16.865	Lilac aldehyde A	1144	MS/RI
23	17.075	(R,S)-5-Ethyl-6-methyl-3E-hepten-2-one	1149	MS/RI
24	17.235	Lilac aldehyde B	1153	MS/RI
25	17.415	(E,Z)-2,6-Nonadienal	1157	MS/RI
26	17.44	Nerol oxide	1157	MS/RI
27	17.9	L-Borneol	1168	MS/RI
28	18.47	Epoxylinalol	1181	MS/RI

29	19.13	α-Terpineol	1196	MS/RI
30	19.29	Methyl salicylate	1199	MS/RI
31	19.435	Safranal	1202	MS/RI
32	20.38	β-Cyclocitral	1222	MS/RI
33	20.925	(Z)-Geraniol	1234	MS/RI
34	21.055	(3Z)-3-Hexenyl 2-methylbutanoate	1237	MS/RI
35	21.255	(Z)-3-Hexenyl isovalerate	1241	MS/RI
36	21.61	Vinyl caproate	1248	MS/RI
37	22.04	β-Cyclohomocitral	1258	MS/RI
38	22.19	Geraniol	1261	MS/RI
39	22.35	(E)-2-Decenal	1264	MS/RI
40	22.84	α-Citral	1274	MS/RI
41	23.445	Anethole	1287	MS/RI
42	23.625	1-Methyl-naphthalene	1291	MS/RI
43	23.825	Theaspirane	1295	MS/RI
44	24.045	Tridecane	1300	MS/RI
45	26.21	Dehydro-ar-ionene	1348	MS/RI
46	26.365	α-Ionene	1351	MS/RI
47	26.695	(Z)-β-Damascenone	1359	MS/RI
48	26.81	2,6,10-Trimethyl-hexadecane	1361	MS/RI
49	27.515	Geranic acid	1377	MS/RI
50	27.675	(E)-β-Damascenone	1381	MS/RI
51	27.87	n-Hexyl caproate	1385	MS/RI
52	28.63	6,10-Dimethyl-2-undecanone	1402	MS/RI
53	29.02	Caryophyllene	1412	MS/RI
54	29.515	α-Ionone	1425	MS/RI
55	29.69	4-(2,4,4-Trimethyl-cyclohexa-1,5-dienyl)-but-3-en-2-one	1429	MS/RI
56	29.94	Dihydro-β-ionone	1435	MS/RI
57	30.635	Geranyl acetone	1453	MS/RI
58	31.295	3-Methyl-tetradecane	1469	MS/RI

59	31.93	β-Ionone	1485	MS/RI
60	32.13	5-Methyl-2-phenyl-2-hexenal	1490	MS/RI
61	32.505	Pentadecane	1500	MS/RI
62	32.86	α-Farnesene	1509	MS/RI
63	33.005	Butylated hydroxytoluene	1513	MS/RI
64	33.225	3,5-Di-t-butylphenol	1518	MS/RI
65	33.605	Dihydroactinidiolide	1528	MS/RI
66	33.865	(-)-Spathulenol	1535	MS/RI
67	35.085	(E)-Nerolidol	1566	MS/RI
68	36.43	Hexadecane	1600	MS/RI
69	37.44	2,6,10-Trimethyl-tetradecane	1627	MS/RI
70	37.58	6-epi-shyobunol	1631	MS/RI
71	38.255	2,6,10-Trimethyl-pentadecane	1649	MS/RI
72	38.6	8-Hexyl-pentadecane	1658	MS/RI
73	39.315	2,2',5,5'-Tetramethyl-1,1'-biphenyl	1677	MS/RI
74	40.15	Heptadecane	1700	MS/RI
75	40.345	2,6,10,14-Tetramethyl-pentadecane	1705	MS/RI
76	43.695	Octadecane	1800	MS/RI
77	45.27	6,10,14-Trimethyl-2-pentadecanone	1855	MS/RI
78	45.625	Caffeine	1868	MS/RI
79	46.065	Diisobutyl phthalate	1884	MS/RI
80	47.82	Farnesyl acetone	1923	MS/RI
81	48.16	Methyl palmitate	1929	MS/RI
82	49.55	Dibutyl phthalate	1953	MS/RI
83	51.14	Ethyl palmitate	1981	MS/RI
84	54.335	Methyl linoleate	2090	MS/RI
85	54.78	Phytol	2108	MS/RI
86	59.1	Squalene	2831	MS/RI

Table S2 The quantitative results of odor-active compounds

Time	Odor-active compounds	Concentration (μg/kg)										
		Sample 1	Sample 2	Sample 3	Sample 4	Sample 5	Sample 6	Sample 7	Sample 8	Sample 9	Sample 10	Sample 11
27.67	(E)-β-Damascenone	125.08	136.42	99.02	238.97	125.52	109.49	69.77	97.13	167.15	58.86	126.46
31.93	β-Ionone	248.43	271.30	317.45	257.71	302.59	244.72	297.74	409.08	297.86	282.99	292.71
29.94	Dihydro-α-ionone	23.80	22.16	12.39	24.15	22.56	18.95	33.86	22.38	25.16	42.08	25.94
15.13	Linalool	2110.46	1577.18	3196.25	2466.72	1930.80	3132.44	1961.21	1609.58	2916.57	900.49	1570.92
29.51	α-Ionone	49.17	38.70	66.41	55.78	71.34	45.64	90.46	113.52	64.94	79.38	66.86
22.19	Geraniol	591.59	172.91	1324.51	1285.33	321.98	206.56	132.79	172.65	364.92	79.47	1103.36
15.75	Phenylethyl Alcohol	393.05	562.85	259.32	286.93	220.34	205.78	223.79	398.09	773.33	244.85	421.37
22.35	(E)-2-Decenal	0.00	0.00	0.00	0.00	147.92	0.00	10.31	122.89	0.00	0.00	0.00
19.29	Methyl salicylate	705.90	1016.74	1418.74	1134.16	523.54	929.48	224.77	375.21	725.78	212.96	1384.81
12.43	Benzeneacetaldehyde	174.98	0.00	52.72	11.86	329.47	219.79	190.66	494.39	680.02	249.92	450.42
35.08	(E)-Nerolidol	241.40	207.64	265.05	259.03	172.47	284.47	141.15	263.99	180.87	0.00	265.00
14.39	Linalool oxide II	981.94	1430.37	862.25	1156.19	672.93	1853.65	853.47	1032.64	1126.53	1077.06	1204.17
9.69	1-Octen-3-ol	9.53	10.11	0.00	9.05	18.19	9.94	178.23	0.00	15.82	0.00	7.84
23.62	1-Methyl-naphthalene	71.48	79.81	67.46	75.28	64.75	0.00	0.00	32.79	254.97	298.42	11.73
20.38	β-Cyclocitral	51.07	0.00	38.17	0.00	87.02	42.22	123.52	90.90	87.29	53.40	63.49
3.845	Hexanal	53.94	69.26	74.19	80.26	44.57	78.33	23.65	164.09	147.63	36.09	76.37
10.05	2-Pentyl-furan	36.13	29.03	17.29	33.48	51.08	49.10	69.98	62.32	54.48	33.56	30.22

13.64	Linalool oxide I	392.87	519.05	352.21	487.96	355.09	1000.30	456.90	493.12	466.65	613.61	466.66
11.56	D-Limonene	57.72	26.52	17.69	29.99	0.00	82.23	45.84	32.04	49.50	28.74	10.83
19.13	α-Terpineol	90.42	76.15	0.00	38.07	39.99	75.09	69.20	18.00	55.72	113.30	0.00
30.63	Geranyl acetone	131.61	84.33	101.15	114.67	108.66	94.74	121.16	126.37	121.64	59.66	116.12
5.32	2-Hexenal	14.61	19.98	81.73	105.40	140.54	0.00	28.96	55.90	90.71	130.01	57.60
33.61	Dihydroactinidiolide	144.28	147.51	249.02	188.89	204.15	125.21	328.21	245.33	0.00	356.32	201.52
17.9	L-Borneol	40.05	57.23	31.05	46.58	8.36	0.00	0.00	38.74	0.00	0.00	82.63
18.47	Epoxylinalol	1049.11	1626.95	495.69	921.55	547.57	1552.48	505.67	1184.75	764.51	927.15	1135.44
23.82	Theaspirane	123.47	110.12	90.19	101.50	98.27	113.05	50.88	41.06	120.02	184.22	99.57
19.43	Safranal	256.32	24.42	0.00	28.50	0.00	0.00	236.66	231.80	0.00	97.17	0.00
8.92	Benzaldehyde	13.68	38.24	12.21	12.94	80.55	93.61	21.29	66.66	156.01	0.00	21.85
11.02	Hexanoic acid	76.32	65.54	75.90	57.70	55.99	0.00	0.00	41.29	148.17	29.63	0.00
20.92	(Z)-Geraniol	44.34	39.33	26.87	8.79	33.12	48.10	22.92	18.36	39.88	17.56	50.56
29.02	Caryophyllene	83.34	100.94	28.46	46.62	0.00	40.12	26.05	0.00	229.91	209.82	56.64
33.01	Butylated Hydroxytoluene	46.41	53.41	38.14	42.45	32.70	22.96	46.92	41.62	48.34	66.99	39.43
27.87	n-Hexyl caproate	114.41	104.49	100.45	116.67	126.64	88.27	108.70	34.50	0.00	0.00	36.48
21.05	(3Z)-3-Hexenyl 2-methylbutanoate	48.33	20.85	45.34	60.83	44.21	40.80	0.00	0.00	59.17	0.00	43.08
27.51	Geranic acid	0.00	0.00	65.96	0.00	117.92	28.67	0.00	0.00	0.00	0.00	63.91

Table S3 The OAVs of odor-active compounds

Time	Odor-active compounds	OAV										
		Sample 1	Sample 2	Sample 3	Sample 4	Sample 5	Sample 6	Sample 7	Sample 8	Sample 9	Sample 10	Sample 11
27.67	(E)-β-Damascenone	62539.41	68208.90	49508.12	119483.38	62760.00	54746.15	34885.00	48563.91	83574.12	29427.88	63229.79
31.93	β-Ionone	35490.69	38756.86	45349.86	36816.08	43227.04	34960.64	42534.41	58440.00	42551.91	40426.71	41815.44
29.94	Dihydro-α-ionone	23798.09	22158.39	12385.95	24154.64	22561.51	18945.12	33863.36	22379.41	25155.64	42081.44	25936.52
15.13	Linalool	351.74	262.86	532.71	411.12	321.80	522.07	326.87	268.26	486.10	150.08	261.82
29.51	α-Ionone	122.93	96.75	166.03	139.45	178.36	114.10	226.15	283.81	162.35	198.46	167.14
22.19	Geraniol	184.87	54.04	413.91	401.67	100.62	64.55	41.50	53.95	114.04	24.83	344.80
15.75	Phenylethyl Alcohol	80.22	114.87	52.92	58.56	44.97	42.00	45.67	81.24	157.82	49.97	85.99
22.35	(E)-2-Decenal	0.00	0.00	0.00	0.00	369.80	0.00	25.77	307.21	0.00	0.00	0.00
19.29	Methyl salicylate	44.12	63.55	88.67	70.89	32.72	58.09	14.05	23.45	45.36	13.31	86.55
12.43	Benzeneacetaldehyde	27.77	0.00	8.37	1.88	52.30	34.89	30.26	78.47	107.94	39.67	71.50
35.08	(E)-Nerolidol	24.14	20.76	26.51	25.90	17.25	28.45	14.12	26.40	18.09	0.00	26.50
14.39	Linalool oxide II	16.37	23.84	14.37	19.27	11.22	30.89	14.22	17.21	18.78	17.95	20.07
9.69	1-Octen-3-ol	6.35	6.74	0.00	6.03	12.13	6.62	118.82	0.00	10.54	0.00	5.23
23.62	1-Methyl-naphthalene	9.53	10.64	8.99	10.04	8.63	0.00	0.00	4.37	34.00	39.79	1.56
20.38	β-Cyclocitral	10.21	0.00	7.63	0.00	17.40	8.44	24.70	18.18	17.46	10.68	12.70
3.845	Hexanal	5.39	6.93	7.42	8.03	4.46	7.83	2.36	16.41	14.76	3.61	7.64
10.05	2-Pentyl-furan	6.02	4.84	2.88	5.58	8.51	8.18	11.66	10.39	9.08	5.59	5.04

13.64	Linalool oxide I	3.93	5.19	3.52	4.88	3.55	10.00	4.57	4.93	4.67	6.14	4.67
11.56	D-Limonene	5.77	2.65	1.77	3.00	0.00	8.22	4.58	3.20	4.95	2.87	1.08
19.13	α-Terpineol	0.21	0.18	0.00	0.09	0.09	0.17	0.16	0.04	0.13	0.26	0.00
30.63	Geranyl acetone	2.19	1.41	1.69	1.91	1.81	1.58	2.02	2.11	2.03	0.99	1.94
5.32	2-Hexenal	0.16	0.22	0.91	1.17	1.56	0.00	0.32	0.62	1.01	1.44	0.64
33.61	Dihydroactinidiolide	0.29	0.30	0.50	0.38	0.41	0.25	0.66	0.49	0.00	0.71	0.40
17.9	L-Borneol	0.29	0.41	0.22	0.33	0.06	0.00	0.00	0.28	0.00	0.00	0.59
18.47	Epoxylinalol	0.35	0.54	0.17	0.31	0.18	0.52	0.17	0.39	0.25	0.31	0.38
23.82	Theaspirane	0.12	0.11	0.09	0.10	0.10	0.11	0.05	0.04	0.12	0.18	0.10
19.43	Safranal	0.26	0.02	0.00	0.03	0.00	0.00	0.24	0.23	0.00	0.10	0.00
8.92	Benzaldehyde	0.02	0.05	0.02	0.02	0.11	0.12	0.03	0.09	0.21	0.00	0.03
11.02	Hexanoic acid	0.09	0.07	0.09	0.06	0.06	0.00	0.00	0.05	0.17	0.03	0.00
20.92	(Z)-Geraniol	0.07	0.06	0.04	0.01	0.05	0.07	0.03	0.03	0.06	0.03	0.07
29.02	Caryophyllene	0.06	0.07	0.02	0.03	0.00	0.03	0.02	0.00	0.15	0.14	0.04
33.01	Butylated Hydroxytoluene	0.05	0.05	0.04	0.04	0.03	0.02	0.05	0.04	0.05	0.07	0.04
27.87	n-Hexyl caproate	0.02	0.02	0.02	0.02	0.02	0.01	0.02	0.01	0.00	0.00	0.01
21.05	(3Z)-3-Hexenyl 2-methylbutanoate	0.00	0.00	0.00	0.01	0.00	0.00	0.00	0.00	0.01	0.00	0.00
27.51	Geranic acid	0.00	0.00	0.01	0.00	0.01	0.00	0.00	0.00	0.00	0.00	0.01

Figure S1

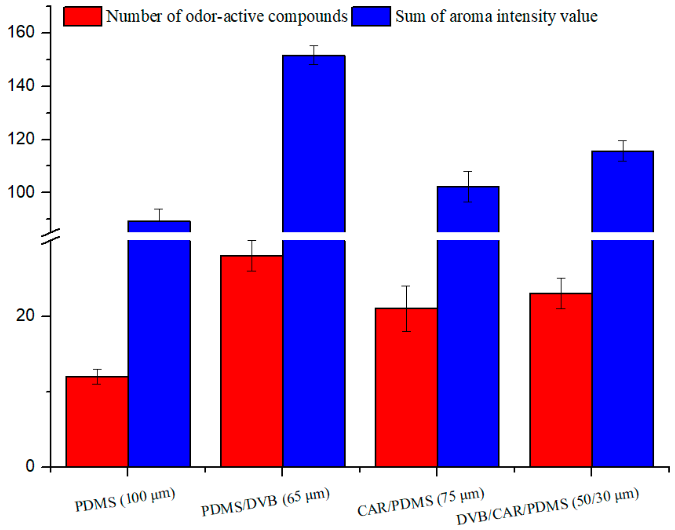

Figure S1 The extraction efficiency of four fibers

Figure S2

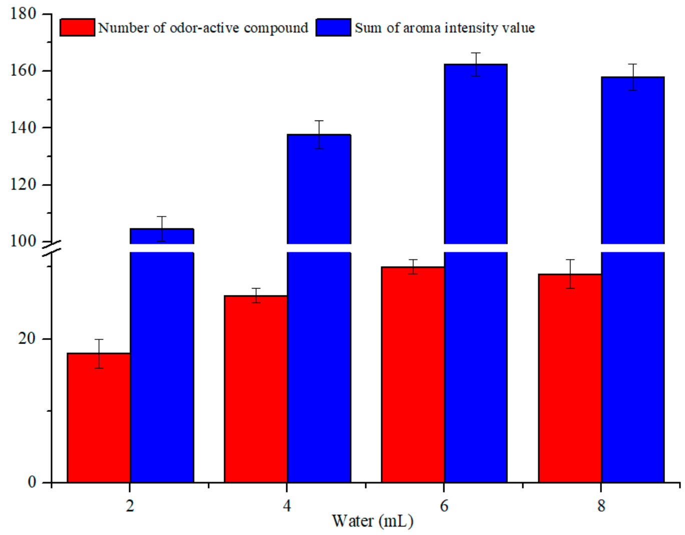

Figure S2 Influence of water amount on the number and sum of intensity value of aroma-active compounds

Figure S3

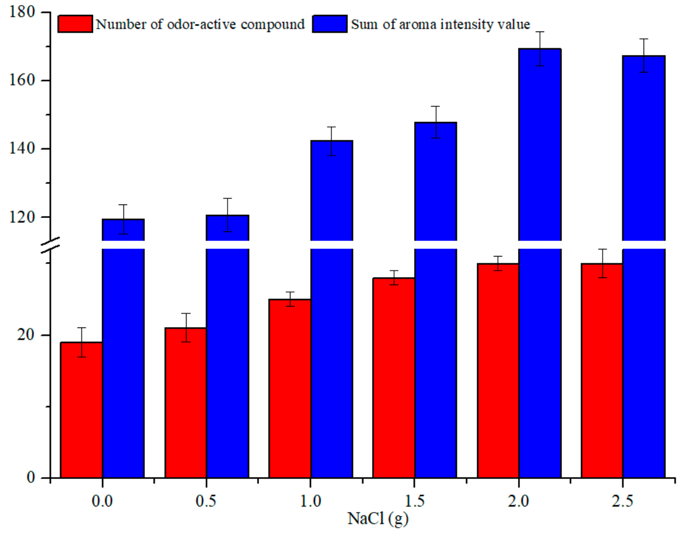

Figure S3 Influence of NaCl amount on the number and sum of intensity value of aroma-active compounds

Figure S4

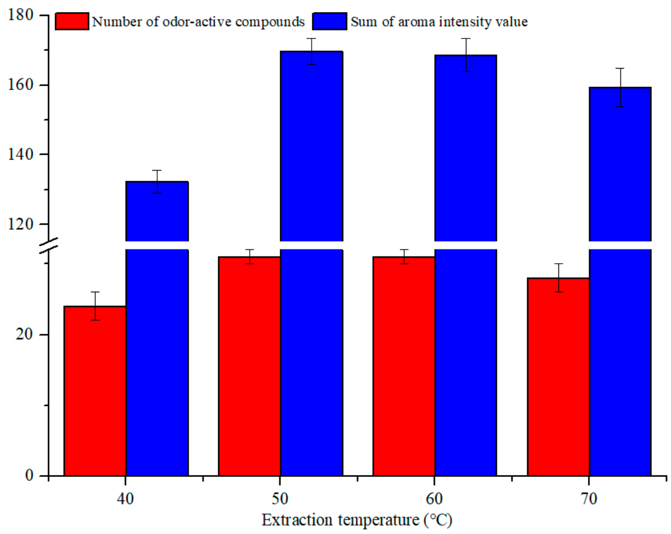

Figure S4 Influence of extraction temperature on the number and sum of intensity value of aroma-active compounds

Figure S5

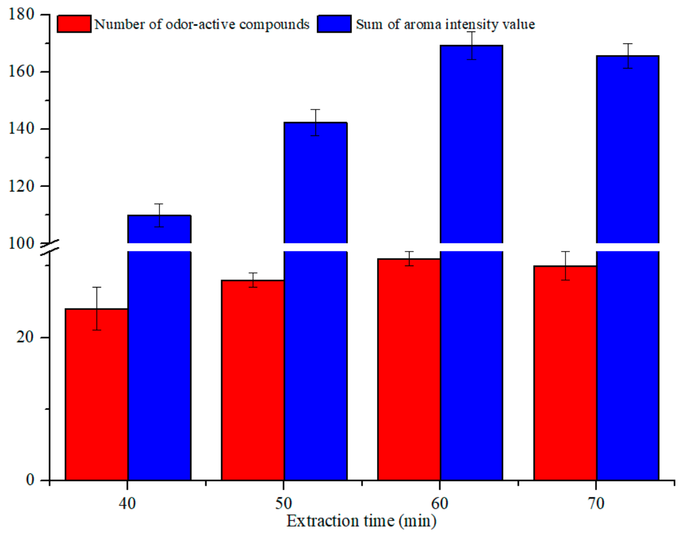

Figure S5 Influence of extraction time on the number and sum of intensity value of aroma-active compounds

Reference

[1] T. Kinoshita, S. Hirata, Z. Yang, S. Baldermann, E. Kitayama, S. Matsumoto, M. Suzuki, P. Fleischmann, P. Winterhalter, N. Watanabe, Formation of damascenone derived from glycosidically bound precursors in green tea infusions. *Food Chem.* 123 (2010) 601-606.

[2] Y. Bezman, I. Bilkis, P. Winterhalter, P. Fleischmann, R.L. Rouseff, S. Baldermann, M. Naim, Thermal oxidation of 9'-cis-neoxanthin in a model system containing peroxyacetic acid leads to the potent odorant β-damascenone. *J. Agric. Food Chem.* 53(2005), 9199-9206.

[3] K. Wang, Y. Wang, Z. Liu, J. Huang, Z. Xu, Y. Li, et al., Comparison of catechins and volatile compounds among different types of tea using high performance liquid chromatograph and gas chromatograph mass spectrometer. *Inter. J. Food Sci Tech*. 46 (2011) 1406-1412.

[4] X. Xu, R. Xu, Q. Jia, T. Feng, Q. Huang, C. Ho, et al. Identification of dihydro-β-ionone as a key aroma compound in addition to C8 ketones and alcohols in Volvariella volvacea mushroom. *Food Chem*. *293* (2019) 333-339.

[5] K. Wang, J.Y. Ruan, Analysis of chemical components in green tea in relation with perceived quality, A case study with Longjing teas. *Inter. J. Food Sci Tech*. 44 (2009) 2476-2484.

[6] M. Wang, W. Ma, J. Shi, Y. Zhu, Z. Lin, H. Lv, Characterization of the key aroma compounds in Longjing tea using stir bar sorptive extraction (SBSE) combined with gas chromatography-mass spectrometry (GC–MS), gas chromatography-olfactometry (GC–O), odor activity value (OAV), and aroma recombination. *Food Res. Inter.* 130 (2020) 108908.

[7] M. Laska, R. Hudson, Comparison of the detection threshold of odour mixtures and their components. *Chemical senses*, *16* (1991).

[8] R.G. Buttery, R.M. Seifert, D.G. Guadagni, D.R. Black, L. Ling, Characterization of some volatile constituents of carrots. *J. Agric. Food Chem.* 16 (1968) 1009-1015.

[9] L.J. van Gemert, (2011). Odour thresholds–Compilations of odour threshold values in air, water and other media. Netherlands: Oliemans Punter & Partners BV, 2003.

[10] X. Pang, W. Yu, C. Cao, X. Yuan, J. Qiu, F. Kong, et al. Comparison of potent odorants in raw and ripened Pu-erh tea infusions based on odor Activity value calculation and multivariate analysis: understanding the role of pile fermentation. *J. Agric. Food Chem.* 67 (2019) 13139-13149.

[11] G.A. Burdock (2010). Fenaroli's handbook of flavor ingredients, sxith ed. CRC Press, Florida.

[12] G.R. Takeoka, R.A. Flath, T.R Mon, R. Teranishi, M. Guentert, Volatile constituents of apricot (Prunus armeniaca). *J. Agric. Food Chem.* 38 (1990) 471-477.

[13] R.G. Buttery, Quantitative and sensory aspects of flavor of tomato and other vegetable and fruits. *Flavor Science: Sensible Principles and Techniques*. (1993).

[14] V. Ferreira, M. Ardanuy, R. Lopez, J. Cacho, Relationship between flavor dilution values and odor unit values in hydroalcoholic solutions: role of volatility and a practical rule for its estimation. *J. Agric. Food Chem.* 46 (1998).

[15] A. Padrayuttawat, T. Yoshizawa, H. Tamura, T. Tokunaga, Optical Isomers and Odor Thresholds of Volatile Constituents in Citrus sudachi. *Food Sci. Tech. Res.* 3 (1997) 402-408.

[16] H. Tamura, R.H. Yang, H. Sugisawa, Aroma profiles of peel oils of acid citrus. *American Chemical Society*, 525 (1993) 121-136.

[17] H. Tamura, S. Boonbumrung, T. Yoshizawa, W. Varanyanond, The Volatile Constituents in the Peel and Pulp of a Green Thai Mango, Khieo Sawoei Cultivar (*Mangifera indica* L.). *Food Sci. Tech Res*, *7* 72-77.
